# Ribosomal Binding Site Switching: An Effective Strategy for High-Throughput Cloning Constructions

**DOI:** 10.1371/journal.pone.0050142

**Published:** 2012-11-21

**Authors:** Yangbo Hu, Lipeng Feng, Yunlong Li, Yong Zhang, Pei Lu, Simon Rayner, Shiyun Chen

**Affiliations:** 1 Key Laboratory of Agricultural and Environmental Microbiology, Wuhan Institute of Virology, Chinese Academy of Sciences, Wuhan, China; 2 Graduate University of the Chinese Academy of Sciences, Beijing, China; Naval Research Laboratory, United States of America

## Abstract

Direct cloning of PCR fragments by TA cloning or blunt end ligation are two simple methods which would greatly benefit high-throughput (HTP) cloning constructions if the efficiency can be improved. In this study, we have developed a ribosomal binding site (RBS) switching strategy for direct cloning of PCR fragments. RBS is an A/G rich region upstream of the translational start codon and is essential for gene expression. Change from A/G to T/C in the RBS blocks its activity and thereby abolishes gene expression. Based on this property, we introduced an inactive RBS upstream of a selectable marker gene, and designed a fragment insertion site within this inactive RBS. Forward and reverse insertions of specifically tailed fragments will respectively form an active and inactive RBS, thus all background from vector self-ligation and fragment reverse insertions will be eliminated due to the non-expression of the marker gene. The effectiveness of our strategy for TA cloning and blunt end ligation are confirmed. Application of this strategy to gene over-expression, a bacterial two-hybrid system, a bacterial one-hybrid system, and promoter bank construction are also verified. The advantages of this simple procedure, together with its low cost and high efficiency, makes our strategy extremely useful in HTP cloning constructions.

## Introduction

The development of the polymerase chain reaction (PCR) represented a major technological breakthrough in the field of molecular biology and genetic engineering. Subsequently, many techniques such as restriction enzyme digestion and ligation [1], ligation-independent cloning (LIC) [2], [3], [4], *in vivo* ligation [5], [6], and site-specific recombination systems [7], [8], [9] have been developed for the cloning of PCR products. However, these methods require either extensive enzymatic treatments of PCR fragments and vectors, or specialized bacterial strains or enzymes. With the development of systematic analyses, e.g., protein interactomes and transcription regulatory networks, there is an increasing demand for high-throughput (HTP) cloning construction methods that can be implemented with simple procedures and at low cost.

Direct cloning of PCR generated fragments by TA cloning or blunt end ligation are two commonly used methods with simple procedures. Fragments generated by Taq polymerase are dAMP tailed and can be directly ligated with T vectors in the presence of T4 DNA ligase [10]. For high flexibility DNA polymerase which contains 3′ to 5′ exonuclease activity, the PCR-generated fragments are blunt ended. Direct ligation of these PCR fragments with blunt end vectors will greatly facilitate the cloning construction [10]. However, despite these features, direct cloning of PCR fragments is not widely applied in HTP cloning constructions because the vector self-ligation and the random orientation of the fragment insertion require additional selection steps. An efficient strategy to eliminate the background from vector self-ligation and reverse insertion of fragments is highly desirable for the application of direct cloning in HTP cloning construction.

There have been a number of technical advances that have improved the efficiency for direct cloning of PCR fragments. One method is to facilitate the clone selection process. In this approach, the reporter gene *lacZ*α, which is used as a marker gene in white/blue colony identification [11], has been replaced by other selectable genes to increase the selection efficiency, such as *gfp* [12], KillerRed gene [13], GST-ParE toxin [14], cytotoxic *ccdB* gene [15], and the *lacZα*-*ccdB* cassette [16]. Among these markers, the *ccdB* gene, whose expression represses bacterial growth by inducing ATP-dependent DNA cleavage [17], greatly facilitates the selection process as only the clones with a PCR fragment insertion can form colonies because of the disruption of CcdB expression. In addition, no substrate is needed in this selection process. However, the problem of determining the orientation of the insertion still needs to be addressed. Strategies which can distinguish the orientation of fragment insertion in direct cloning have been reported, these include digestion of ligated products by enzymes before transformation to remove fragments inserted in the reverse orientation [18]; insertion of 5′ end tailed fragments at the 3′ end of a mutated *bla* or *cat* gene to restore their activity which can select the clones with a fragment inserted in the forward orientation [19]; and 3′ end tailed fragments with a region covering an active RBS to the translational start codon ATG can be inserted upstream of the un-translated *lacZα* gene in the vector, thus the forward insertion will be specifically selected by *lacZα* expression [20]. Nevertheless, these methods have limited applications due to the additional steps in the cloning procedure or requirement for a special insertion site in the vector, and have not been applied to HTP cloning constructions.

In prokaryotes, the ribosomal binding site (RBS) is an A/G rich sequence that is 5–7 nucleotides upstream of the translational start codon [21]. The A/G content is important for gene expression. A change from A/G to T/C can significantly decrease or even block RBS activity [22]. Based on this property, we developed a new strategy named RBS switching for direct cloning of PCR fragments. This approach incorporates an insertion site within an inactive RBS such that forward insertion of specific tailed PCR fragments into the vector can form an active RBS (high A/G content), and vector self-ligation and fragment reverse insertions will form low A/G inactive RBS. Thus, all background will be eliminated during the clone selection process, and this is confirmed for TA cloning and blunt end ligations using this strategy. Application of our strategy to gene over-expression, bacterial two-hybrid systems, bacterial one-hybrid systems, and promoter bank construction are also verified. The simplicity of this procedure, together with its low cost and high efficiency, gives our strategy widespread application in systematic analyses that require HTP cloning constructions.

## Materials and Methods

### RBS Activities Confirmation

**Figure 1:**
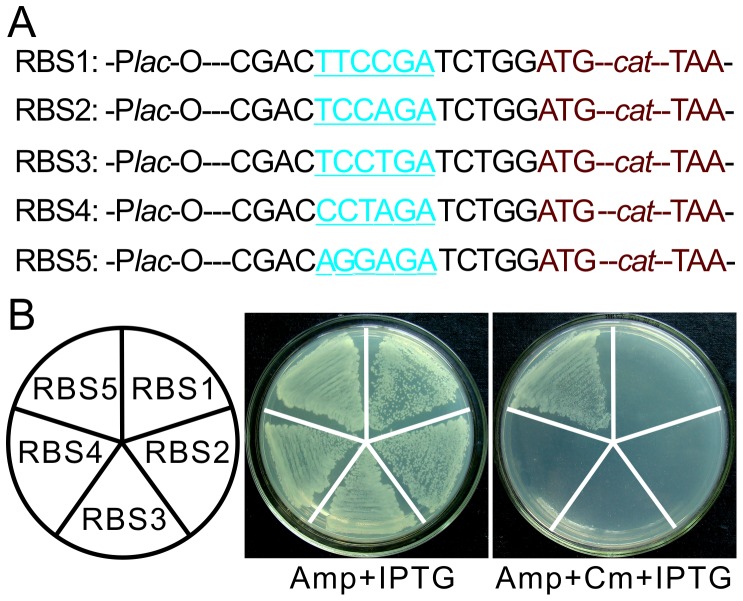
Influence of A/G content in the RBS sequence on gene expression. (**A**) Design of five different RBS sequences (blue with underline) upstream of the *cat* gene (brown). All fragments were inserted downstream of the *lac*-O promoter (P*lac*-O) in pMD19-T vector and named RBS1 to RBS5. (**B**) Analysis of activities of these RBS sequences by Cm resistance. Five *E. coli* recombinant strains with plasmids containing RBS1 to RBS5 shown in left were respectively streaked on LB plates containing Amp+IPTG or Amp+Cm+IPTG.

Primers for PCR amplification of the *cat* gene are listed in **Table S1**. Each forward primer contains an RBS sequence upstream of the *cat* open reading frame (ORF) as shown in **Figure 1A**. Five *cat* fragments containing different RBSs were amplified from the pACYC184 plasmid (NEB) using KOD DNA polymerase (TOYOBO) and treated with Taq polymerase to add dAMP to the 3′ end, and were respectively ligated into the pDM19T vector (TAKARA). Clones containing the *cat* gene inserted in the same direction as the *lac*-O promoter in the vector were selected by PCR and confirmed by sequencing. These clones were spread onto LB agar plates containing either ampicillin (Amp) and isopropyl β-D-thiogalactoside (IPTG), or Amp, chloramphenicol (Cm) and IPTG to confirm the expression of the *cat* gene.

### pDNB100 Construction

The procedure for construction of pDNB100 is detailed in **Figure S1**. Briefly, primers PDNB-KF and PDNB-BR (**Table S1**) were designed to amplify the backbone of a vector named as pDNB-A, which contains the *lac* promoter, the pUC ori, and the *bla* gene from the pUC18 plasmid (TAKARA). The promoterless *cat* gene was amplified from the pACYC184 plasmid using primers Pcat-BF and Pcat-KR (**Table S1**). PCR fragments of *cat* and pDNB-A were both digested with *Bgl*II and *Kpn*I and ligated to form vector pDNB-B. The *Xcm*I cassette, which contains a large spacer DNA and a negative selection marker *ccdB*, was generated as described in the supplementary data and inserted between the *EcoR*I and *Bgl*II sites in the pDNB-B plasmid to obtain vector pDNB100, and was transformed into *E. coli* DB3.1 (Invitrogen), which is capable of expression of CcdB in the *Xcm*I cassette.

### TA Cloning

The plasmid pDNB100 was treated with *Xcm*I to create T tails on the linearized plasmid to obtain pDNB100-T. Primers for *lacZ*α and *gfpuv* amplifications are listed in **Table S1**. The forward primer contains an active RBS sequence and an additional AGG at the 5′ end, the reverse primer contains an additional CCT at the 5′ end. These fragments (0.075 pM) were amplified by Taq polymerase and ligated into pDNB100-T (0.025 pM) by T4 DNA ligase. The resulting plasmids were transformed into *E. coli* DH5α and spread onto LB plates containing either Amp or Amp and Cm to compare the selection efficiency. 5-bromo-4-chloro-3-indolyl-D-galactopyranoside (X-gal) and IPTG were added to LB medium (named as LBXI) to examine the expression of the *lacZ*α gene by α-complementation in β-galactosidase activity. Primers paired to the 5′ end of the *lac* promoter in pDNB100-T and the 3′ end of inserted fragments were applied to confirm the forward insertion of PCR fragments.

### Blunt End Cloning

For blunt end ligation, the pDNB100-T vector was first treated with T4 DNA polymerase in the presence of dNTPs. For the 3′ to 5′ exonuclease and 5′ to 3′ polymerase activities from T4 DNA polymerase, the dT tails in pDNB100-T vector were removed in this process to obtain a blunt end vector pDNB100-B. Primers for PCR fragments are listed in **Table S1**, which are the same as the primers used in TA cloning except that TAGG was added to the 5′ end of each forward primer and TCCT was added to the 5′ end of each reverse primer. The RBS-containing *lacZ*α and *gfpuv* fragments (0.075 pM) were amplified by proof-reading DNA polymerase (KOD DNA polymerase) and then ligated with pDNB100-B (0.025 pM) by T4 DNA ligase. All these constructs were transformed into *E. coli* DH5α and spread onto LB plates with Amp or Amp plus Cm. LBXI plates were also used to confirm the expression of the *lacZ*α gene. For cloning the *lacZ*α gene into the pDNB101 vector, AGG was introduced to the 5′ end of forward primer upstream of the ATG start codon and CCT was introduced to the 5′ end of the reverse primer. For cloning the *lac* promoter fragment (P*lac*) into pDNB107, the forward primer was tailed with sequence CCTCTCTTCAAATAATTATATCACA, which contains a reverse complementary TGnTATAAT promoter (named as “−10 extended promoter” and shown as underlined) to initiate the *cat* gene transcription after insertion into the vector. The reverse primer was tailed with AGG. PCR was applied to confirm the forward insertion of PCR fragments in these constructions.

## Results

### A/G Content in the RBS Determines the Gene Expression

To confirm the importance of A/G content for gene expression in the RBS sequence, we amplified five *cat* fragments with different RBS sequences (difference in A/G content is shown in **Figure 1A**), which were then inserted into the pMD19T vector in the same orientation with the *lac*-O promoter (**Figure 1A**). To test the function of these five RBS sequences, expression of the *cat* gene (which encodes chloramphenicol acetyltransferase) was detected by Cm resistance in the presence of IPTG to induce the transcription initiated by the *lac*-O promoter. As shown in **Figure 1B**, all of the five clones grew well on LB plates without Cm, but only the clone containing RBS5 could grow on the plate with Cm, which means only the RBS5 can initiate the translation of the *cat* gene among these RBS sequences. Based on these observations, we proposed a strategy for clone construction in which a PCR fragment is inserted into an inactive RBS sequence with suitable A/G content upstream of the *cat* gene. If the PCR fragment is tailed with specific nucleotides, it will allow the formation of active RBS only when the fragment is inserted in the forward orientation. We therefore term this strategy "RBS switching". Since an inactive RBS cannot initiate the *cat* translation, the background caused by vector self-ligation and reverse insertion of fragments will be removed when clone selection is performed on Cm plates.

### Vector Construction Incorporating the RBS Switching in Clone Selection

**Figure 2:**
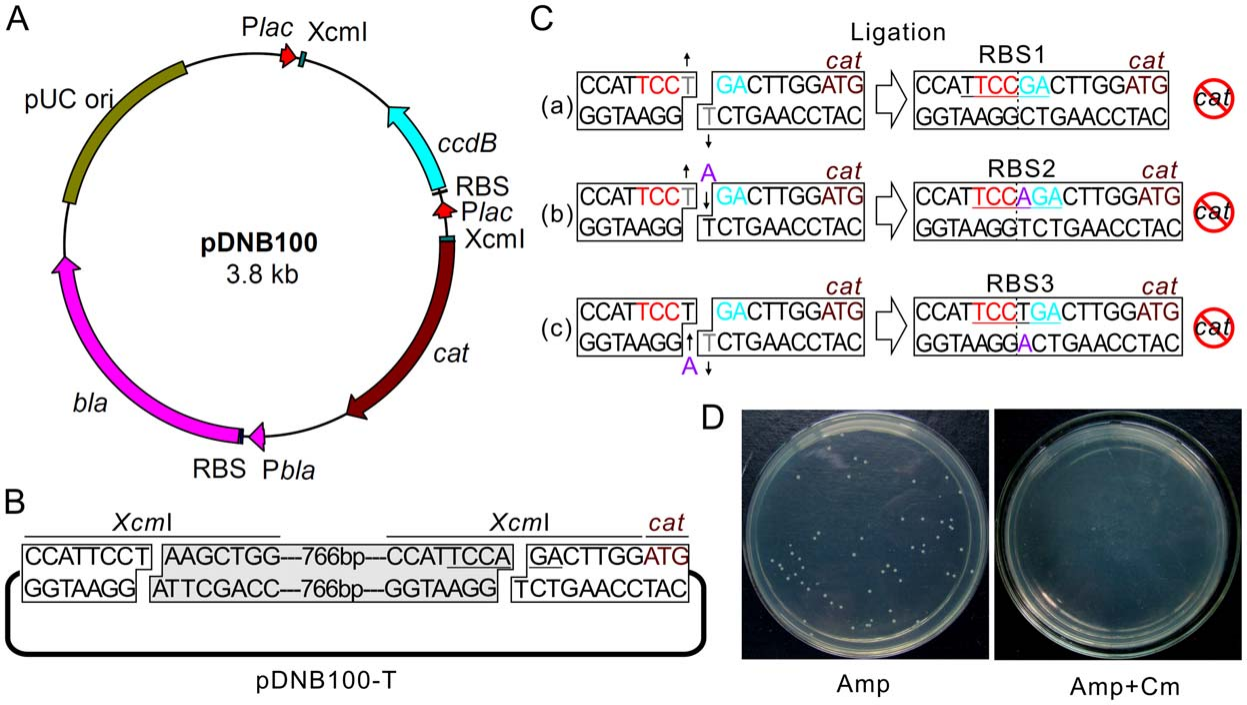
Vector map of pDNB100 and its efficiency in removing vector self-ligation. (**A**) Vector map of pDNB100. All key elements are labeled with different colors. (**B**) Frame of pDNB100-T. T tails are created by *Xcm*I digestion of pDNB100 plasmid. The removed fragment is shown by the grey box. (**C**) Different situations for self-ligation of the pDNB100-T vector. The grey “T” represents the nucleotide that been removed during the ligation step or after been transformed into *E. coli*. The purple “A” represents nucleotide added to the plasmid by the bacterial DNA repair system. The start codon ATG for the *cat* gene is shown in brown. More details are given in the main text. (**D**) Transformants of pDNB100-T self-ligation product on LB plates with (right) or without (left) Cm.

To further prove our hypothesis, we constructed a vector named pDNB100 which contained the *lac* promoter, the pUC ori, the *bla* and *cat* genes, and an *Xcm*I cassette (with two *Xcm*I sites) between the *lac* promoter and the *cat* gene (**Figure 2A** **)**. The *ccdB* negative selectable marker gene was introduced into the *Xcm*I cassette to facilitate the vector preparation [23]. This vector contains two *Xcm*I sites in the *Xcm*I cassette (**Figure 2B** **)**, which will create double T tails and form a T vector for TA cloning after digestion with *Xcm*I. The right-hand *Xcm*I site upstream of the *cat* gene contains an inactive RBS sequence (TCCAGA). Digestion with *Xcm*I will cut between the A and G inside of this inactive RBS so that insertion of specific tailed PCR fragments or vector self-ligation will form new RBS sequences. The left *Xcm*I site is T/C rich, thus self-ligation of this vector after it has been digested with *Xcm*I will form an inactive RBS. The *lac* promoter in this vector does not contain the O site, so that its activity is not inhibited by the LacI protein and it is not necessary to add IPTG in the clone selection.

### Application of RBS Switching Strategy in TA Cloning

The efficiency of our RBS switching strategy in TA cloning was tested first. To do this, the pDNB100 plasmid was digested with *Xcm*I to obtain the T vector named pDNB100-T. There are three possible outcomes for the self-ligation of this vector as shown in **Figure 2C**: (I). the two T tails are both removed to produce blunt ends which are used to form RBS1 upstream of the *cat* gene after self-ligation; (II). ligation takes place between the ends on the lower strand, and the unpaired T on the upper strand is repaired *in vivo* to form RBS2 after transformation; (III). similar to II, but the ligation takes place between the ends on the upper strand to form RBS3. All these RBS sequences have been demonstrated to be inactive as shown in **Figure 1**. Accordingly, transformants with self-ligation of pDNB100-T formed colonies on plates without Cm but could not form colonies containing Cm (**Figure 2D**).

**Figure 3:**
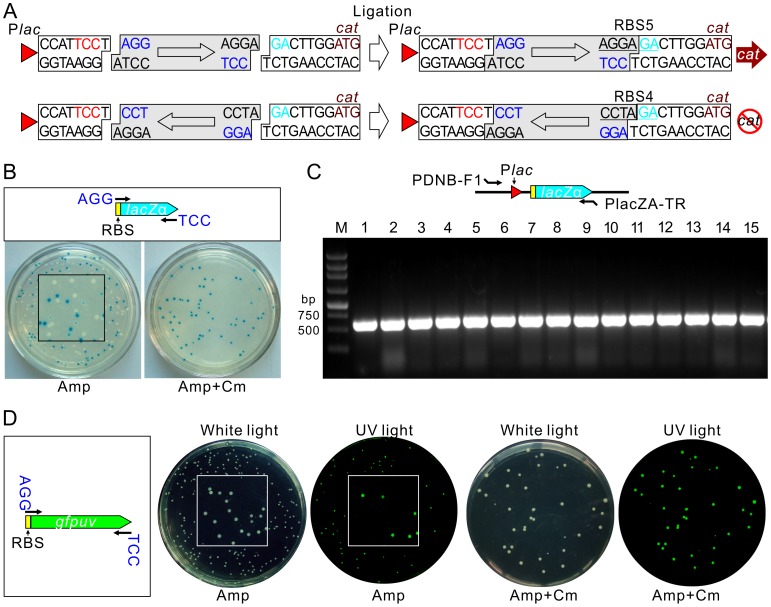
Application of pDNB100-T in TA cloning construction. (**A**) RBS sequences formed by forward and reverse insertion of PCR fragment. The boxed arrowhead indicates the orientation of the PCR fragment. The purple nucleotides are introduced by primers. (**B**) Cloning of a RBS containing a *lacZα* fragment. The upper panel shows the scheme for PCR amplification. Transformants on plate without (left) or with (right) Cm are shown. Expression of *lacZα* in *E. coli* DHα is detected by formation of blue/white colonies using X-gal as substrate and in the presence of IPTG. The square in the center of plate shows a magnified image. (**C**) PCR confirmation of blue colonies on plate containing Cm as shown in B. Paired positions for primers used in PCR are shown in the upper panel. (**D**) Cloning of a RBS containing a *gfpuv* fragment. The scheme for PCR amplification is also shown in the left panel. Transformants of ligated products on plates without or with Cm under white light and ultraviolet (UV) light are shown to illustrate the expression of GFPuv.

Having established the background from self-ligation can be eliminated in our strategy, the next question was whether it is possible to distinguish the orientation of PCR fragment insertion. Since RBS activity is sequence dependent, we introduced AGG to the 5′ end of the forward primer and CCT to the reverse primer. As shown in **Figure 3A**, the forward insertion of the PCR fragment generated by Taq polymerase will create an active RBS upstream of the *cat* gene (RBS5), while a reverse insertion will form an inactive RBS (RBS4). To test this, we designed a pair of primers (**Table S1)** for the *lacZα* fragment by which an active RBS was introduced to initiate the expression of *lacZα*. The PCR fragment was directly ligated with pDNB100-T and transformed into *E. coli* DH5α, which can be used in α-complementation in β-galactosidase analysis. Only the forward insertion of *lacZα* into pDNB100-T can restore the β-galactosidase activity in *E. coli* DH5α and this forms blue colonies on LBXI plates since there is no promoter to initiate the transcription of *lacZα* when it is reverse inserted. To test the effectiveness of this cloning construction, we spread the transformants onto LBXI plates in the absence or presence of Cm. As shown in **Figure 3B**, colonies are in white or blue on plates without Cm, which means these colonies are mixtures of clones with forward or reverse insertions of *lacZα*, or self-ligation of the vector. However, all colonies are in blue on the plates with Cm, which indicates the RBS switching strategy has successfully removed the background from vector self-ligation and the reverse insertion of PCR fragments. To confirm the forward insertion of *lacZα* into pDNB100-T on the LBXI plates with Cm, we randomly selected 15 colonies and tested by PCR using a forward primer paired to the *lac* promoter and a reverse primer paired to the 3′ end of *lacZα*. As expected, a 580 bp PCR product was amplified from all of these colonies (**Figure 3C**).

Insertion of a RBS containing a *gfpuv* fragment into pDNB100-T was also examined in a similar way to the *lacZα* experiment. As shown in **Figure 3D**, less than half of the colonies can be detected with fluorescence under UV light on LB plates without Cm, while application of the Cm plate to the clone selection process significantly increased the efficiency, as demonstrated by the fact that all of the colonies on plates with Cm were detected with fluorescence. Taken together, these results demonstrate that our RBS switching is an efficient strategy for TA cloning as all background is eliminated during selection.

### Application of RBS Switching Strategy in Blunt End Ligation

**Figure 4:**
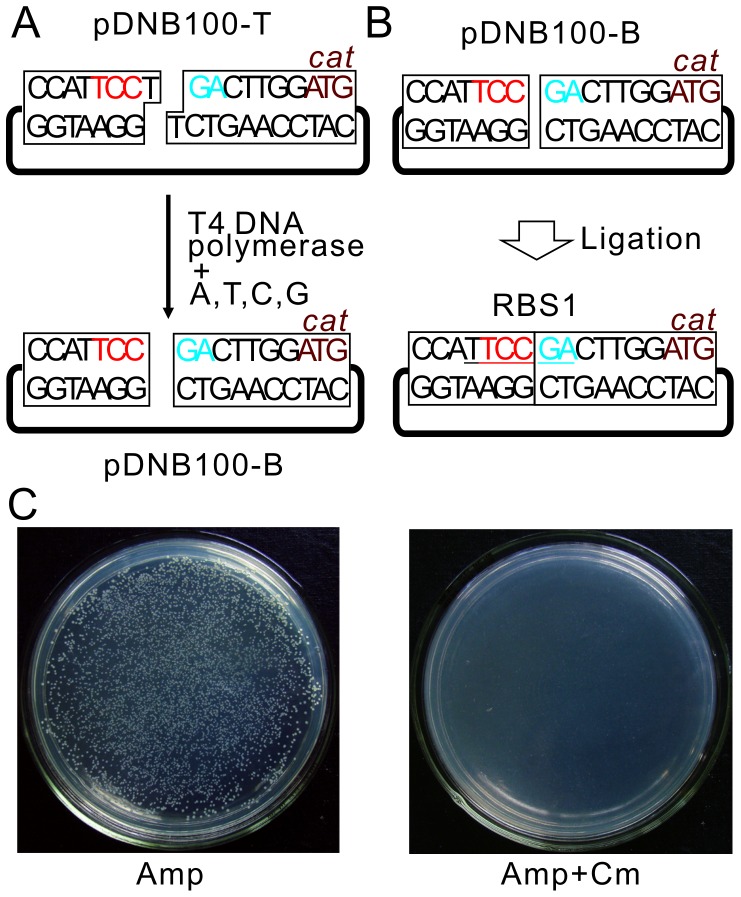
Overview of pDNB100-B preparation and its efficiency at eliminating self-ligation. (**A**) Scheme for pDNB100-B vector construction. The T tails in pDNB100-T are treated with T4 DNA polymerase in the presence of four nucleotides. (**B**) Situation for pDNB100-B self-ligation. RBS sequence formed by vector self-ligation is underlined. (**C**) Transformants of pDNB100-B self-ligation product on LB plates in the absence (left) or presence of Cm (right).

For high-fidelity DNA polymerase generated PCR fragments, blunt end ligation is an easier way to make clone constructions compared to TA cloning. However, self-ligation is a major problem with the blunt end ligation method. Having shown the RBS switch strategy can totally remove the background in TA cloning, we next examined the efficiency of this strategy applied to blunt end ligation. To do this, we treated the pDNB100-T with T4 DNA polymerase in the presence of dNTPs to remove the dT tails in the plasmid and obtained a new vector named pDNB100-B (**Figure 4A**). In this case, there is only one scenario for the self-ligation of this vector which is to form RBS1 upstream of the *cat* gene (**Figure 4B**). The RBS1 sequence was already demonstrated to be inactive (**Figure 1**), so self-ligation for pDNB100-B can be removed by adding Cm. As shown in **Figure 4C**, transformants grew well on plates without Cm, but no colony was detected on plates with Cm.

**Figure 5:**
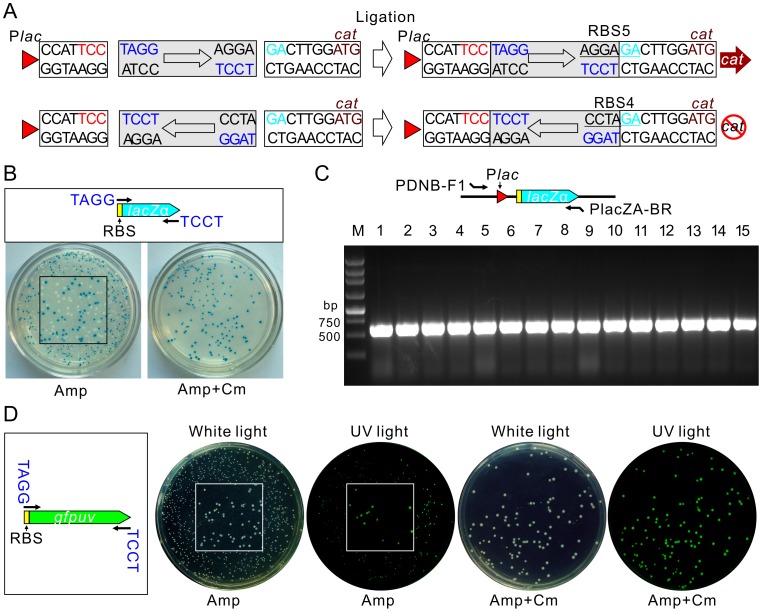
Application of pDNB100-B in blunt end cloning construction. (**A**) RBS sequences formed by forward and reverse insertion of PCR fragments. The boxed arrowhead indicates the orientation of PCR fragments. Initiation codon ATG for the *cat* gene is shown in brown. (**B**) Application of pDNB100-B in cloning of a RBS containing a *lacZα* fragment. The upper panel shows the scheme for PCR amplification. Transformants of ligated products on plate without (left) or with (right) Cm are shown. The expression of *lacZα* is detected by β-galactosidase activity. (**C**) PCR confirmation of blue colonies as shown in part B. Paired positions for primers used in PCR are shown in the upper panel. (**D**) Cloning of a RBS-containing *gfpuv* fragment using pDNB100-B vector. The scheme for PCR amplification is also shown in the left panel. Transformants of ligated products are spread onto plates with or without Cm to compare the efficiency of *cat* selection. Images under white or UV light are also compared to test the expression of GFPuv. The squares in the center of plate images show an enlarged section of the image.

To distinguish the orientation of PCR fragment insertions, we designed primers with additional nucleotides. The primers used here were distinct from the TA cloning primers in that the forward primer contained TAGG (instead of AGG in TA cloning) and the reverse primer contained TCCT (instead of CCT in TA cloning). The forward and reverse insertions of PCR fragments generated by proof-reading polymerase into pDNB100-B will form RBS5 and RBS4, respectively (**Figure 5A**), which are in consistent with the corresponding situations in TA cloning. Thus, we would expect to obtain a similar efficiency with application of pDNB100-B in blunt end ligation.

Cloning of the blunt end *lacZα* and *gfpuv* fragments into pDNB100-B were also tested. TAGG and TCCT tailed primers (PlacZA-BF and PlacZA-BR listed in **Table S1**) were designed to amplify the blunt end RBS containing the *lacZα* fragment. The ligated products were transformed into *E. coli* DH5α and were spread onto plates with or without Cm. We observed white and blue colonies on plates without Cm but, as expected, all colonies were blue on plates with Cm (**Figure 5B**). PCR was then employed to confirm the forward insertion of the *lacZα* fragment and all of the 15 randomly selected colonies from the Cm plate were positive (**Figure 5C**). The same experiment was also carried out for the RBS containing a *gfpuv* fragment. As shown in **Figure 5D**, only a part of colonies can be detected with fluorescence under UV light on plates without Cm, while all colonies are detectable by fluorescent in the presence of Cm. Together, these results demonstrate that the RBS switching strategy is also a highly effective method for elimination of the vector self-ligation and the reverse insertion of PCR fragments in blunt end cloning.

### Application of the RBS Switching Strategy in Different Cloning Constructions

**Figure 6:**
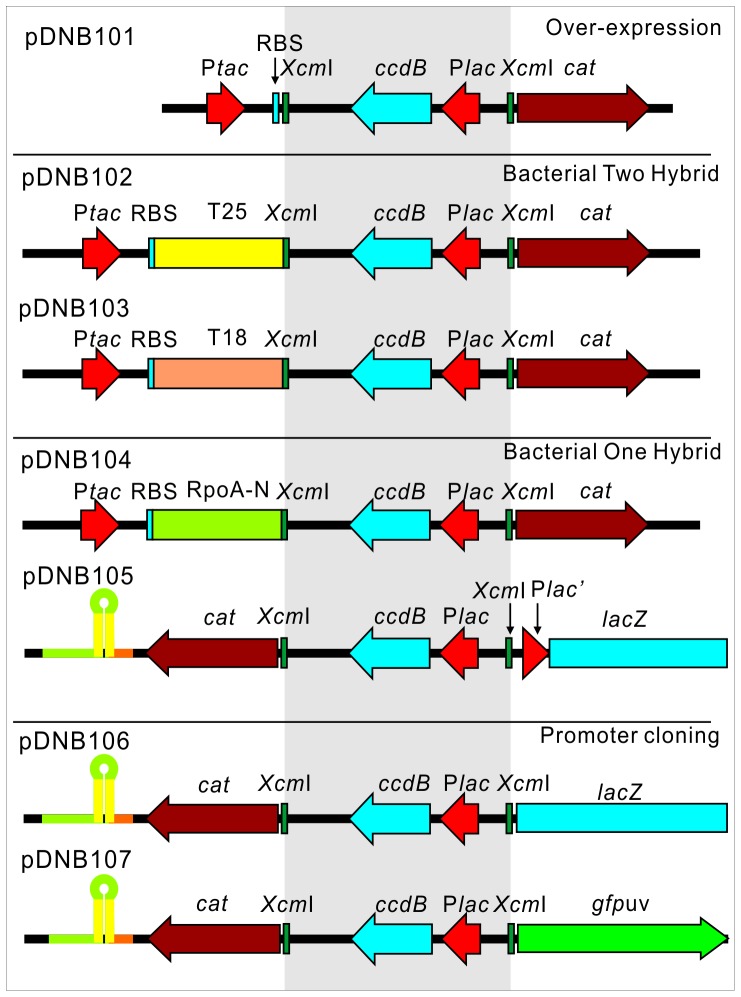
Schematic of plasmids incorporating the RBS switching strategy in different cloning constructions. Plasmids for gene over-expression (pDNB101), bacterial two-hybrid system (pDNB102 and pDNB103), bacterial one-hybrid system (pDNB104 and pDNB105) and promoter bank construction (pDNB106 and pDNB107) are presented. Each key element in the vector is labeled and a strong terminator upstream of the *cat* reporter gene in each of pDNB105, pDNB106 and pDNB107 is shown as a hairpin structure. The *Xcm*I cassette in each plasmid is shown with grey background.

Having demonstrated the high efficiency of the RBS switching strategy in TA and blunt end cloning of PCR fragments, we next examined this strategy applied to other clone constructions. We were particularly interested in the application to HTP cloning constructions, e.g., protein over-expression library construction, protein-protein interactome assays, transcription regulatory network analyses and promoter screening. To facilitate these constructions, we designed several blunt end cloning vectors to incorporate the RBS switching strategy in clone selection (**Figure 6**, details can be found in **Figure S2**). For overexpression vectors comprising plasmids pDNB101 to pDNB104, a similar strategy as used for pDNB100 was applied but the *lac* promoter was replaced by a stronger *tac* promoter to increase the expression level of the genes of interest. As shown in **Figure 6** **,** the adenylate cyclase dependent bacterial two-hybrid system [24] and the RpoA dependent bacterial one-hybrid system [23] were applied to facilitate large scale protein interaction assays and transcriptional regulatory network analyses. For vectors used for promoter cloning in a bacterial one-hybrid system (pDNB105) or promoter bank constructions (pDNB106 and pDNB107), a reporter gene (*lacZ* or *gfpuv*) was first introduced, and then the *cat* selectable marker was inserted in the reverse direction upstream of the reporter gene. Finally, the *XcmI* cassette was introduced between the *cat* gene and the reporter gene so that the RBS switching for the *cat* gene in these plasmids will take place at the left-hand *Xcm*I site (**Figure 6**). A weak P*lac*’ promoter (a mutated *lac* promoter) was designed between the *Xcm*I cassette and the *lacZ* reporter to maintain low background expression of the reporter gene in pDNB105 [23]. There is no promoter to initiate the transcription of the *cat* gene in these vectors after they been blunt end treated (**Figure 6**). An additional reverse complement "−10 extended promoter" sequence CCTCTCTTCAAATAATTATATCACA (the promoter sequence is underlined) needs to be introduced to the forward primer, as opposed to the three or four nucleotides in the other constructions, in order to drive the transcription of the *cat* gene during the clone screening.

The efficiencies of these vectors in cloning construction are demonstrated by the results for pDNB101 (for gene expression) and pDNB107 (for promoter cloning). The RBS sequence upstream of the *cat* gene in each of these gene overexpression plasmids (pDNB101-pDNB104) was modified such that AGA will be left after blunt end treatment (as opposed to GA in pDNB100-B) as shown in **Figure S2B**. Thus, only three additional nucleotides (AGG or CCT) are needed for the RBS recombination instead the four required in the pDNB100-B vector (**Figure 5**). To obtain the correct expression, the inserted fragments need to be in the same frame as the translational coding frame derived from these plasmids. Considering this, we designed primers for the open reading frame (ORF) of the *lacZα* gene and introduced AGG to the 5′ end of the forward primer and CCT to 3' end of the reverse primer (**Table S1**). Fragments amplified by proof-reading polymerase were inserted into blunt end treated pDNB101. As shown in **Figure S3A**, all colonies were in blue on LBXI plates with Cm, which suggests that the *lacZα* gene is inserted into pDNB101 in the forward direction; this was then confirmed by PCR for 15 randomly selected colonies as shown in **Figure S3A**. Cloning of the *lac* promoter (P*lac*) into pDNB107, which contains a promoterless *gfpuv* gene as the reporter gene downstream of the insertion site, was also tested. As stated above, we introduced a reverse complementary "−10 extended promoter" to the forward primer (**Table S1**). The PCR fragment was directly ligated with the blunt end treated pDNB107 vector and the transformants were spread onto LB plates with Cm. As shown in **Figure S3B**, all colonies on the Cm plate are clones with forward insertion of the P*lac* fragments as demonstrated by both fluorescence test and PCR. Taken together, these results demonstrate that the RBS switching strategy was successfully applied in these vectors for systematic analyses.

## Discussion

TA cloning and blunt end ligation are two standard methods for direct DNA cloning with simple procedures requiring simple protocols [10]. Both of these methods could potentially be applied to HTP cloning constructions if their efficiency could be improved. For example, although application of TA cloning in promoter bank construction of *Mycobacterium tuberculosis* for bacterial one-hybrid analysis has been developed [23], it has only seen limited usage in large scale cloning constructions due to restrictions in efficiency. For this reason, the large scale cloning constructions in the subsequent transcription regulatory analysis in this bacterium were carried out by restriction enzyme digestion and ligation rather than TA cloning [25]. In this study, we have developed a strategy based on RBS switching to control the expression of a selectable marker gene such that all background caused by vector self-ligations and fragment reverse insertions are eliminated by clone selection. Thus, only clones with forward insertion of PCR fragments from colonies remain, which greatly simplifies the clone selection procedure and provides opportunities for application to systematic analysis requiring HTP cloning constructions.

An RBS sequence usually contains a polypurine domain found just 5′ to the translational start codon [21]. It is now clear that the RBS sequence is important for identification of the translation initiation site by the ribosome. As a result, the sequence of RBS, the spacing between the RBS and the initiation codon, and the secondary structure of RBS around mRNA all strongly affect translational efficiency [22], [26]. The riboswitch, which generates the change in structure of mRNA around the RBS sequence in order to control gene translation, has now been widely used in different gene expression systems [27], [28]. However, there is no report applying RBS sequence recombination to control the expression of a selectable gene in clone selection. Here we tested the effect of replacement of A/G by C/T in the first four nucleotides in RBS and observed that this abolished the translation of the *cat* gene in our system (**Figure 1**). Based on these observations, we designed a RBS switching strategy which implements RBS sequence recombination to control RBS activity upstream of a selectable marker gene.

Besides its high efficiency, there are several advantages of our RBS switching strategy in cloning constructions. First, this strategy can be applied in both TA cloning and blunt end ligation. Blunt end ligation is compatible with proof-reading DNA polymerases, which makes it easy to obtain error-free DNA. Thus, it is a better choice than TA cloning for high fidelity cloning of PCR fragments. However, self-ligation of normal blunt end vectors is a major problem that needs to be resolved for the effective application of this method. Dephosphorylation of blunt vectors can reduce self-ligation to some extent but this requires that the PCR fragment be phosphorylated by additional treatment or by phosphorylated primers [29], which increases the cost and/or complexity of the cloning construction. Because of this, direct cloning of the PCR fragment by TA ligation is preferred [23]. In this study, we applied the RBS switching strategy in clone selection so that self-ligation of the vector forms an inactive RBS that represses the expression of the selectable marker gene. The blunt end of the vector designed in this study has not been dephosphorylated so that the PCR fragment does not need any additional treatment, and the procedure for blunt end ligation becomes as simple as TA cloning but with a significant increase in the fidelity of cloning.

The second advantage is that there is no limitation for the length of PCR fragments to be cloned. Classical selection by the *lacZα* gene or its derivatives is based on insertional inactivation [11]. Insertion of some fragments, especially short fragments, might not completely disrupt expression of the reporter gene, making it difficult to distinguish a colony carrying plasmids with or without an insert. Our RBS switching is dependent on the RBS sequence, which is recombined by fragment insertion and is strictly dependent on the insertion of a fragment that forms the active RBS, but not on the fragment length. In this study, we have shown the efficiency of pDNB100 in cloning fragments between 250 bp and 700 bp (**Figure 3** **&** **5**). Insertion of a 45 bp fragment generated by primers annealing into pDNB100-B has also been tested and all of the 20 randomly selected colonies from plates with Cm were tested with forward insertion of fragment by PCR as shown in **Figure S4**.

Probably the most attractive feature of our strategy is its potential for widespread application in cloning constructions. Although some strategies for direct cloning of PCR fragments with distinguishable orientation have been reported for normal T-vectors [19], [20], there has been no demonstration of their application in promoter cloning or gene over-expression in systematic analysis. As shown in **Figure 6**, we have designed different vectors for gene over-expression and promoter cloning including a bacterial two-hybrid system, a bacterial one-hybrid system, and promoter bank constructions based on previous publications [23], [24]. Only blunt end ligation is demonstrated since the efficiency for blunt cloning is higher than TA cloning in our system (109 colonies in blunt end cloning in **Figure 5D** compared with 38 colonies in **Figure 3D** with Cm selection). Also, the fidelity of proof-reading DNA polymerase (used in blunt end ligation) is much higher than Taq polymerase (used in TA cloning). For vectors pDNB100-T or pDNB100-B, different fragments can be cloned. Normally we only need to introduce three or four nucleotides to each end of the PCR fragments as stated above. But the RBS switching might be disrupted when we clone a fragment with strong terminator which may stop the transcription initiated from the *lac* promoter in the vector upstream of *cat* gene. Under this condition, we need to introduce a −10 promoter instead of only three nucleotides to the reverse primer, so that transcription of the *cat* gene will be initiated by the −10 promoter and the RBS switching can also be successfully used in a similar way as the application of pDNB107 in cloning promoter fragment. In addition to the vectors described in this study, many more vectors can be modified in a similar way to incorporate our RBS switching strategy in cloning selection to meet the requirements for different HTP cloning constructions.

For vectors pDNB100 to pDNB107, we used the *cat* gene as the selectable marker, it is reasonable to expect that the *cat* gene can be replaced by any other positive selection marker genes. Negative selection markers, such as *ccdB* [15] and *sacB* [30], can also be used but the RBS switching needs to be reversed so that only the forward insertion of PCR fragments can lead to the inactive RBS formation. In this way, self-ligation or reverse insertion of the PCR fragments will both form an active RBS which will initiate the expression of these negative selection marker genes to repress bacterial growth. Negative selection marker genes are especially useful in gene over-expression cloning constructions due to the unwanted expression of marker genes together with cloned fragments. But it is important to ensure that the expression of these negative selection genes in vector self-ligation and fragment reverse insertion is high enough to repress bacterial growth. Also, *Xcm*I is used but is not the only choice for the fragment insertion site. Other restriction enzymes can be used by inserting alternate recognition sites in the RBS sequence upstream of the selectable marker genes.

Although all these vectors need to be treated before cloning, the treated vector can be used in cloning different kinds of fragments and stored at −20°C for use before cloning. Furthermore, the PCR fragment does not need any special kind of treatment, which greatly simplifies the cloning procedure. With all of these features, we conclude that the strategy developed in this study has widespread application in fragment cloning and is especially useful in HTP cloning constructions in systematic analyses.

## Supporting Information

Figure S1 **Flow chart for pDNB100 construction.** Primers PDNB-KF and PDNB-BR (Table S1) were used to amplify pDNB-A, which contains the *lac* promoter, the pUC *ori* and the *bla* gene from the pUC18 plasmid. The promoterless *cat* gene was amplified from pACYC184 using primers Pcat-BF and Pcat-KR (Table S1), which was digested with *Bgl*II and *Kpn*I and ligated into pDNB-A to obtain pDNB-B. A RBS-containing *ccdB* gene was amplified from *E. coli* XL-blue using primers PccdB-EF and PccdB-KR (Table S1), digested with *EcoR*I and *Kpn*I and inserted into pDNB-A to obtain pDNB-ccdB. Two primers containing the *Xcm*I sites (PXcmI-EF and PXcmI-BR in Table S1) were designed to amplify the fragment covering the region from the *lac* promoter to the *ccdB* gene in pDNB-ccdB. Primers (POF and POR in Table S1) were used for overlapping PCR to eliminate the *EcoR*I site to obtain the *Xcm*I cassette, which was then digested with *EcoR*I and *Bgl*II and inserted into pDNB-B to obtain pDNB100.
(TIF)

Figure S2 **Details for pDNB101 to pDNB107.** (**A**) Plasmid maps of pDNB101 to pDNB107 and their applications. The *tac* promoter in pDNB101 to pDNB104 plasmids is followed by the LacI binding site to decrease the expression of inserted genes in the absence of IPTG. An active RBS and ATG translational start codon or T18 (24), T25 (24) and RpoA-N (23) peptides was respectively introduced upstream of the *Xcm*I cassette in pDNB101 to pDNB104 to drive the translation of inserted genes. The *cat* gene was introduced in the opposite direction in pDNB105 to pDNB107 compared with pDNB101 to pDNB104. Two compatible replicate origins (pMB1 and P15A) were introduced into these plasmids to meet the requirements for the two-plasmid systems (bacterial two-hybrid system (24) and one-hybrid system (23)). A weak P*lac*’ promoter (a mutant *lac* promoter) was designed between the *Xcm*I cassette and the *lacZ* reporter to maintain low background expression of the reporter gene in pDNB105. (**B**) *Xcm*I sites of pDNB101 to pDNB104. The start codon for the *cat* gene is indicated in brown and the two *Xcm*I sites are shown in green. The frame of reading codon from the initiation ATG in pDNB101 or peptides in pDNB102 to pDNB104 is indicated by a horizontal line. The blunt end treated product from pDNB101 to pDNB104 is shown in the lower panel. RBS sequences upstream of the *cat* gene in these plasmids and after vector self-ligation are underlined. (**C**) *Xcm*I sites of pDNB105 to pDNB107. The start codon of *cat* on the opposite strand is shown in brown. RBS sequences on the opposite strand are underlined.
(TIF)

Figure S3 **Application of pDNB101 and pDNB107 in cloning constructions.** (**A**) Application of pDNB101 in cloning the *lacZ*α fragment. RBS sequences formed by forward and reverse insertions of PCR fragments are indicated in blue and underlined. Colonies on the LBXI plate containing Cm are shown. Fifteen randomly selected colonies were tested by PCR to verify the orientation of fragment insertion. The primer paired positions used in PCR are shown by arrowheads. (**B**) Cloning of the *lac* promoter (P*lac*) into pDNB107. RBS sequences formed by forward and reverse insertion of PCR fragment are indicated. Colonies on a plate containing Cm are shown under white and UV light to illustrate the expression of GFPuv in pDNB107. Ten colonies were tested by PCR to confirm the orientation of fragment insertion using primers paired to the 5′ end of P*lac* and the 3′ end of the *gfpuv* gene.
(TIF)

Figure S4 **Application of pDNB100-B in cloning a 45 bp fragment.** (**A**) RBS sequences formed by forward and reverse insertion of the 45 bp fragment. The arrows indicate the orientation of the inserts. The ATG start codon for the *cat* gene is shown in brown. (**B**) Transformants of ligated products on plate with Cm. (C) PCR confirmation of colonies as shown in B. Paired positions for primers used in PCR are shown in the upper panel.
(TIF)

Table S1 Oligonucleotides used in this study.
(DOC)

## References

[1] Scharf SJ, Horn GT, Erlich HA (1986) Direct cloning and sequence analysis of enzymatically amplified genomic sequences. Science 233: 1076–1078. 3461561 10.1126/science.3461561

[2] Aslanidis C, de Jong PJ (1990) Ligation-independent cloning of PCR products (LIC-PCR). Nucleic Acids Res 18: 6069–6074. 2235490 10.1093/nar/18.20.6069 PMC332407

[3] Hsiao K (1993) Exonuclease III induced ligase-free directional subcloning of PCR products. Nucleic Acids Res 21: 5528–5529. 8265374 10.1093/nar/21.23.5528 PMC310601

[4] Nour-Eldin HH, Hansen BG, Norholm MH, Jensen JK, Halkier BA (2006) Advancing uracil-excision based cloning towards an ideal technique for cloning PCR fragments. Nucleic Acids Res 34: e122. 17000637 10.1093/nar/gkl635 PMC1635280

[5] Bubeck P, Winkler M, Bautsch W (1993) Rapid cloning by homologous recombination in vivo. Nucleic Acids Res 21: 3601–3602. 8346047 10.1093/nar/21.15.3601 PMC331480

[6] Oliner JD, Kinzler KW, Vogelstein B (1993) In vivo cloning of PCR products in *E. coli* . Nucleic Acids Res 21: 5192–5197. 8255776 10.1093/nar/21.22.5192 PMC310636

[7] Bushman W, Thompson JF, Vargas L, Landy A (1985) Control of directionality in lambda site specific recombination. Science 230: 906–911. 2932798 10.1126/science.2932798 PMC1978455

[8] Hartley JL, Temple GF, Brasch MA (2000) DNA cloning using in vitro site-specific recombination. Genome Res 10: 1788–1795. 11076863 10.1101/gr.143000 PMC310948

[9] Fu C, Wehr DR, Edwards J, Hauge B (2008) Rapid one-step recombinational cloning. Nucleic Acids Res 36: e54. 18424799 10.1093/nar/gkn167 PMC2396420

[10] D'Arpa P (2009) Strategies for cloning PCR products. Cold Spring Harb Protoc 2009: pdb ip68. 10.1101/pdb.ip68 20147231

[11] Takeshita S, Sato M, Toba M, Masahashi W, Hashimoto-Gotoh T (1987) High-copy-number and low-copy-number plasmid vectors for lacZ alpha-complementation and chloramphenicol- or kanamycin-resistance selection. Gene 61: 63–74. 3327753 10.1016/0378-1119(87)90365-9

[12] Miura H, Inoko H, Inoue I, Tanaka M, Sato M, et al (2011) Simple cloning strategy using GFPuv gene as positive/negative indicator. Anal Biochem 416: 237–239. 21601558 10.1016/j.ab.2011.04.040

[13] Liu X, Liu X, Zhou Y, Zou D, Shi R, et al (2010) T vector bearing KillerRed protein marker for red/white cloning screening. Anal Biochem 405: 272–274. 20599648 10.1016/j.ab.2010.06.031

[14] Kim HG, Kim HS, Hwang HJ, Chung SK, Lee JM, et al (2004) Construction of a pTOC-T vector using GST-ParE toxin for direct cloning and selection of PCR products. Biotechnol Lett 26: 1659–1663. 15604816 10.1007/s10529-004-3518-z

[15] Bernard P, Gabant P, Bahassi EM, Couturier M (1994) Positive-selection vectors using the F plasmid *ccdB* killer gene. Gene 148: 71–74. 7926841 10.1016/0378-1119(94)90235-6

[16] Gabant P, Dreze PL, Van Reeth T, Szpirer J, Szpirer C (1997) Bifunctional *lacZα-ccdB* genes for selective cloning of PCR products. Biotechniques 23: 938–941. 9383562 10.2144/97235pf01

[17] Bernard P, Kezdy KE, Van Melderen L, Steyaert J, Wyns L, et al (1993) The F plasmid CcdB protein induces efficient ATP-dependent DNA cleavage by gyrase. J Mol Biol 234: 534–541. 8254658 10.1006/jmbi.1993.1609

[18] Horn D (2005) Directional enrichment of directly cloned PCR products. Biotechniques 39: 40, 42, 44, 46. 10.2144/05391BM03 16060367

[19] Malo MS, Husain Z (2003) Positive selection vectors for high-fidelity PCR cloning. Biotechniques 34: 1250–1258. 12813893

[20] Keese P, Graf L (1996) A positive screen for cloning PCR products. Nucleic Acids Res 24: 3474–3475. 8811107 10.1093/nar/24.17.3474 PMC146101

[21] Shine J, Dalgarno L (1975) Determinant of cistron specificity in bacterial ribosomes. Nature 254: 34–38. 803646 10.1038/254034a0

[22] Vellanoweth RL, Rabinowitz JC (1992) The influence of ribosome-binding-site elements on translational efficiency in *Bacillus subtilis* and *Escherichia coli in vivo* . Mol Microbiol 6: 1105–1114. 1375309 10.1111/j.1365-2958.1992.tb01548.x

[23] Guo M, Feng H, Zhang J, Wang W, Wang Y, et al (2009) Dissecting transcription regulatory pathways through a new bacterial one-hybrid reporter system. Genome Res 19: 1301–1308. 19228590 10.1101/gr.086595.108 PMC2704442

[24] Karimova G, Pidoux J, Ullmann A, Ladant D (1998) A bacterial two-hybrid system based on a reconstituted signal transduction pathway. Proc Natl Acad Sci U S A 95: 5752–5756. 9576956 10.1073/pnas.95.10.5752 PMC20451

[25] Zeng J, Cui T, He ZG (2012) A Genome-wide Regulator-DNA Interaction Network in the human pathogen *Mycobacterium tuberculosis* H37Rv. J Proteome Res 11: 4682–4692. 22808930 10.1021/pr3006233

[26] Chen H, Bjerknes M, Kumar R, Jay E (1994) Determination of the optimal aligned spacing between the Shine-Dalgarno sequence and the translation initiation codon of *Escherichia coli* mRNAs. Nucleic Acids Res 22: 4953–4957. 7528374 10.1093/nar/22.23.4953 PMC523762

[27] Topp S, Reynoso CM, Seeliger JC, Goldlust IS, Desai SK, et al (2010) Synthetic riboswitches that induce gene expression in diverse bacterial species. Appl Environ Microbiol 76: 7881–7884. 20935124 10.1128/AEM.01537-10 PMC2988590

[28] Seeliger JC, Topp S, Sogi KM, Previti ML, Gallivan JP, et al (2012) A riboswitch-based inducible gene expression system for mycobacteria. PLoS One 7: e29266. 22279533 10.1371/journal.pone.0029266 PMC3261144

[29] Sambrook J, Russell DW (2006) Blunt-end Cloning of PCR Products. CSH Protoc 2006. 10.1101/pdb.prot3830 22485323

[30] Blomfield IC, Vaughn V, Rest RF, Eisenstein BI (1991) Allelic exchange in *Escherichia coli* using the *Bacillus subtilis sacB* gene and a temperature-sensitive pSC101 replicon. Mol Microbiol 5: 1447–1457. 1686293 10.1111/j.1365-2958.1991.tb00791.x

