# Supplementary figures and images for: Ribosomal Binding Site Switching: An Effective Strategy for High-Throughput Cloning Constructions
Source: PLoS One. 2012 Nov 21;7(11):e50142. doi: 10.1371/journal.pone.0050142 (PMC3503710; doi:10.1371/journal.pone.0050142)

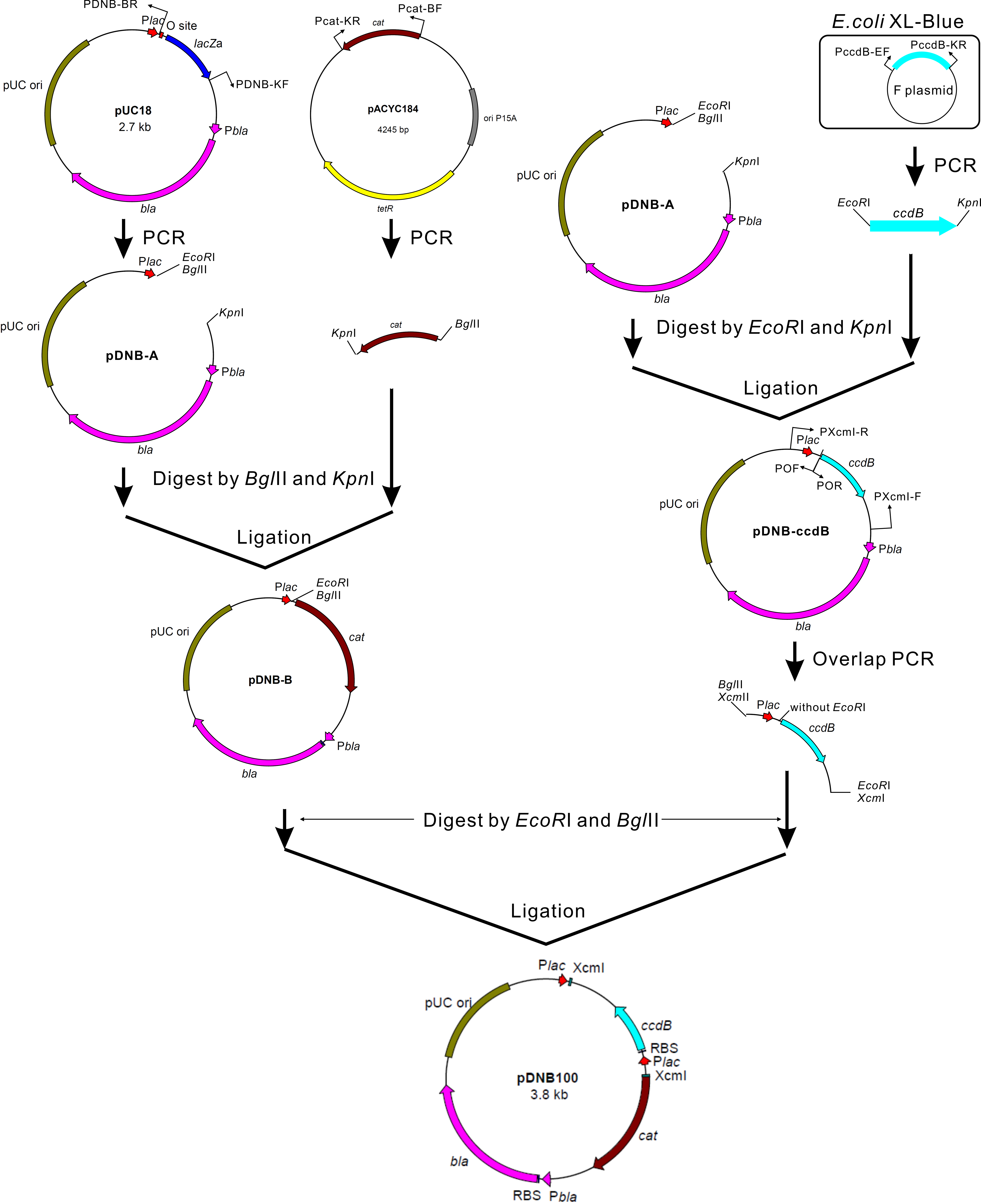

Supplement: Figure S1 — Flow chart for pDNB100 construction. Primers PDNB-KF and PDNB-BR (Table S1) were used to amplify pDNB-A, which contains the lac promoter, the pUC ori and the bla gene from the pUC18 plasmid. The promoterless cat gene was amplified from pACYC184 using primers Pcat-BF and Pcat-KR (Table S1), which was digested with BglII and KpnI and ligated into pDNB-A to obtain pDNB-B. A RBS-containing ccdB gene was amplified from E. coli XL-blue using primers PccdB-EF and PccdB-KR (Table S1), digested with EcoRI and KpnI and inserted into pDNB-A to obtain pDNB-ccdB. Two primers containing the XcmI sites (PXcmI-EF and PXcmI-BR in Table S1) were designed to amplify the fragment covering the region from the lac promoter to the ccdB gene in pDNB-ccdB. Primers (POF and POR in Table S1) were used for overlapping PCR to eliminate the EcoRI site to obtain the XcmI cassette, which was then digested with EcoRI and BglII and inserted into pDNB-B to obtain pDNB100. (TIF) [file pone.0050142.s001.tif]

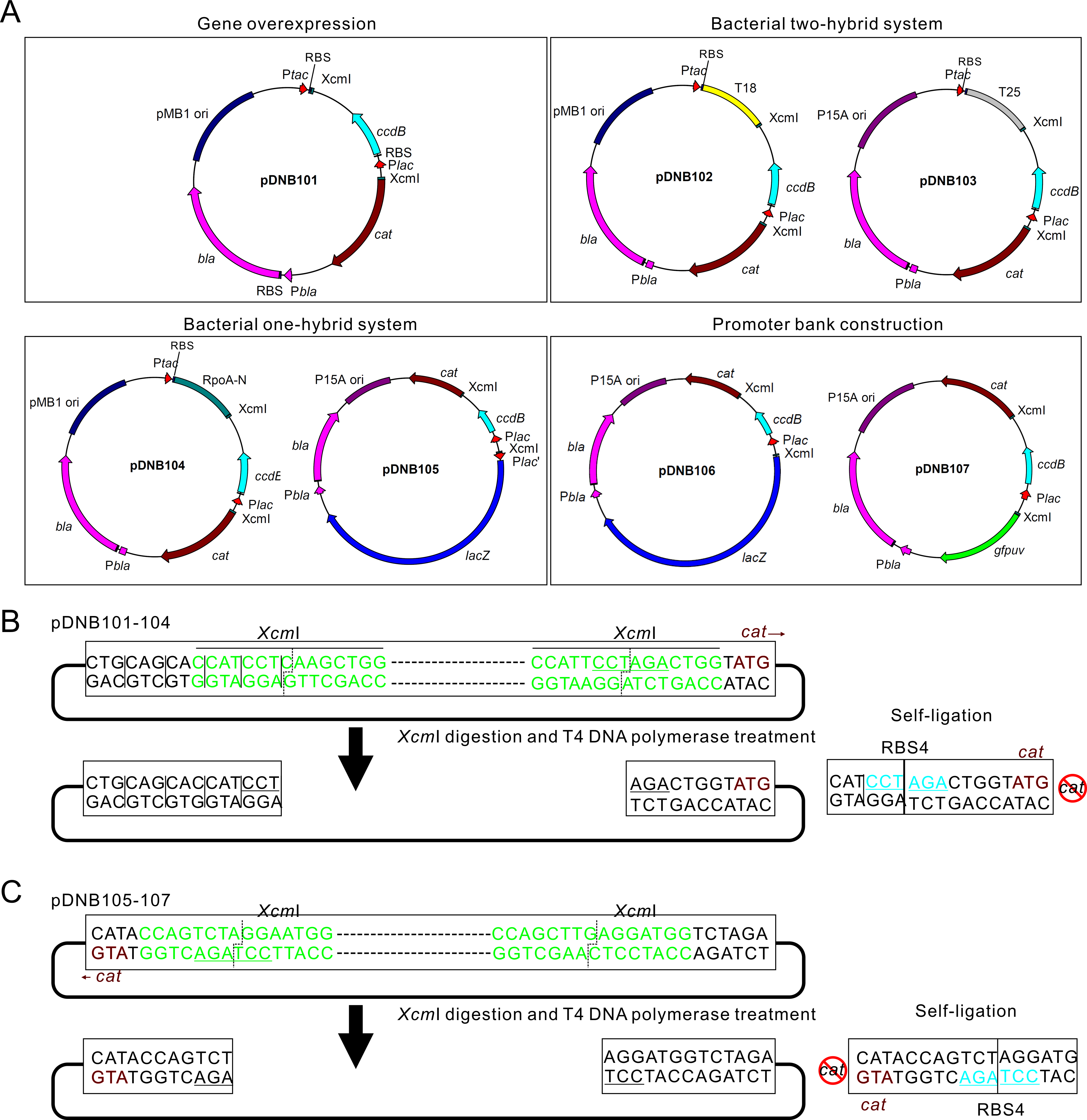

Supplement: Figure S2 — Details for pDNB101 to pDNB107. (A) Plasmid maps of pDNB101 to pDNB107 and their applications. The tac promoter in pDNB101 to pDNB104 plasmids is followed by the LacI binding site to decrease the expression of inserted genes in the absence of IPTG. An active RBS and ATG translational start codon or T18 (24), T25 (24) and RpoA-N (23) peptides was respectively introduced upstream of the XcmI cassette in pDNB101 to pDNB104 to drive the translation of inserted genes. The cat gene was introduced in the opposite direction in pDNB105 to pDNB107 compared with pDNB101 to pDNB104. Two compatible replicate origins (pMB1 and P15A) were introduced into these plasmids to meet the requirements for the two-plasmid systems (bacterial two-hybrid system (24) and one-hybrid system (23)). A weak Plac’ promoter (a mutant lac promoter) was designed between the XcmI cassette and the lacZ reporter to maintain low background expression of the reporter gene in pDNB105. (B) XcmI sites of pDNB101 to pDNB104. The start codon for the cat gene is indicated in brown and the two XcmI sites are shown in green. The frame of reading codon from the initiation ATG in pDNB101 or peptides in pDNB102 to pDNB104 is indicated by a horizontal line. The blunt end treated product from pDNB101 to pDNB104 is shown in the lower panel. RBS sequences upstream of the cat gene in these plasmids and after vector self-ligation are underlined. (C) XcmI sites of pDNB105 to pDNB107. The start codon of cat on the opposite strand is shown in brown. RBS sequences on the opposite strand are underlined. (TIF) [file pone.0050142.s002.tif]

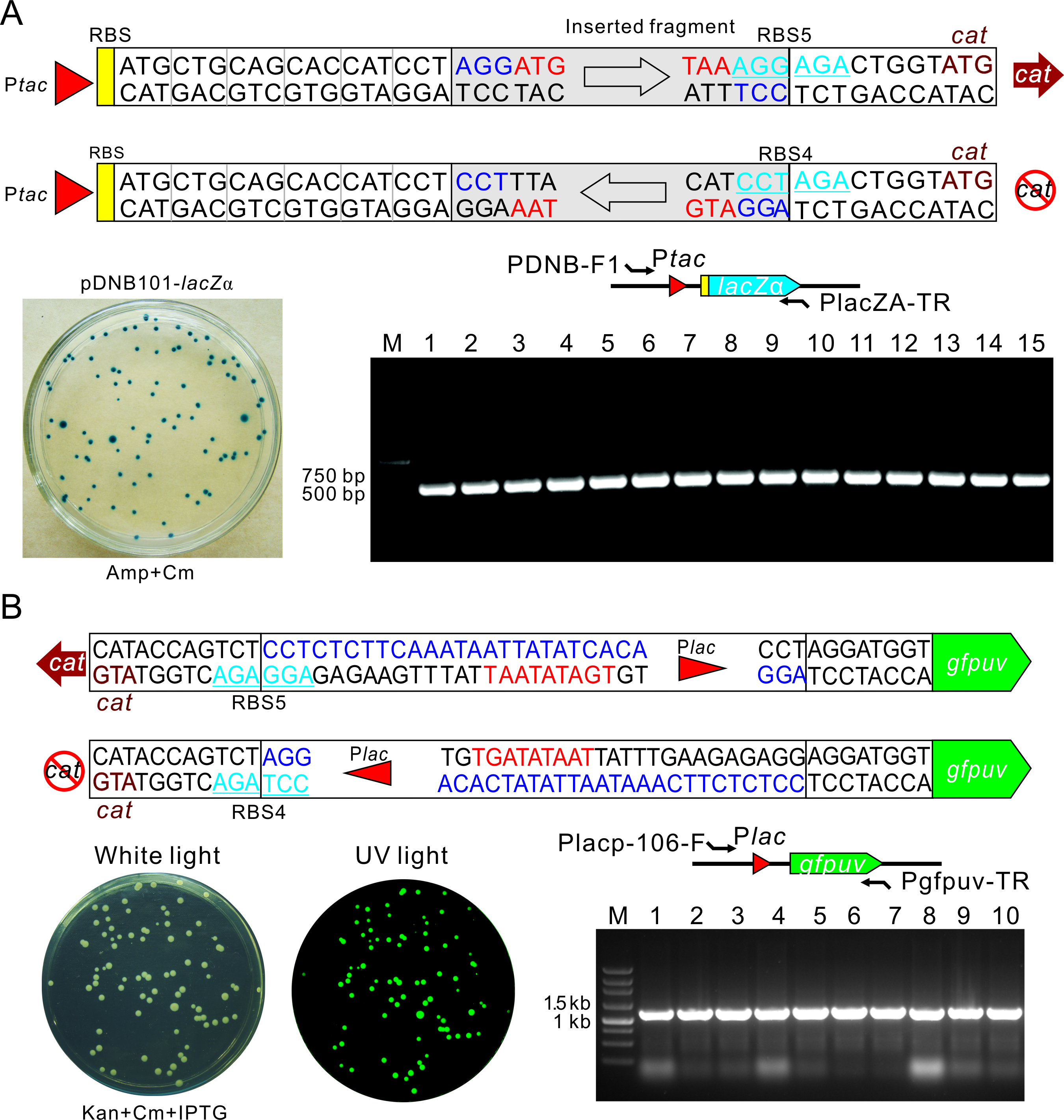

Supplement: Figure S3 — Application of pDNB101 and pDNB107 in cloning constructions. (A) Application of pDNB101 in cloning the lacZα fragment. RBS sequences formed by forward and reverse insertions of PCR fragments are indicated in blue and underlined. Colonies on the LBXI plate containing Cm are shown. Fifteen randomly selected colonies were tested by PCR to verify the orientation of fragment insertion. The primer paired positions used in PCR are shown by arrowheads. (B) Cloning of the lac promoter (Plac) into pDNB107. RBS sequences formed by forward and reverse insertion of PCR fragment are indicated. Colonies on a plate containing Cm are shown under white and UV light to illustrate the expression of GFPuv in pDNB107. Ten colonies were tested by PCR to confirm the orientation of fragment insertion using primers paired to the 5′ end of Plac and the 3′ end of the gfpuv gene. (TIF) [file pone.0050142.s003.tif]

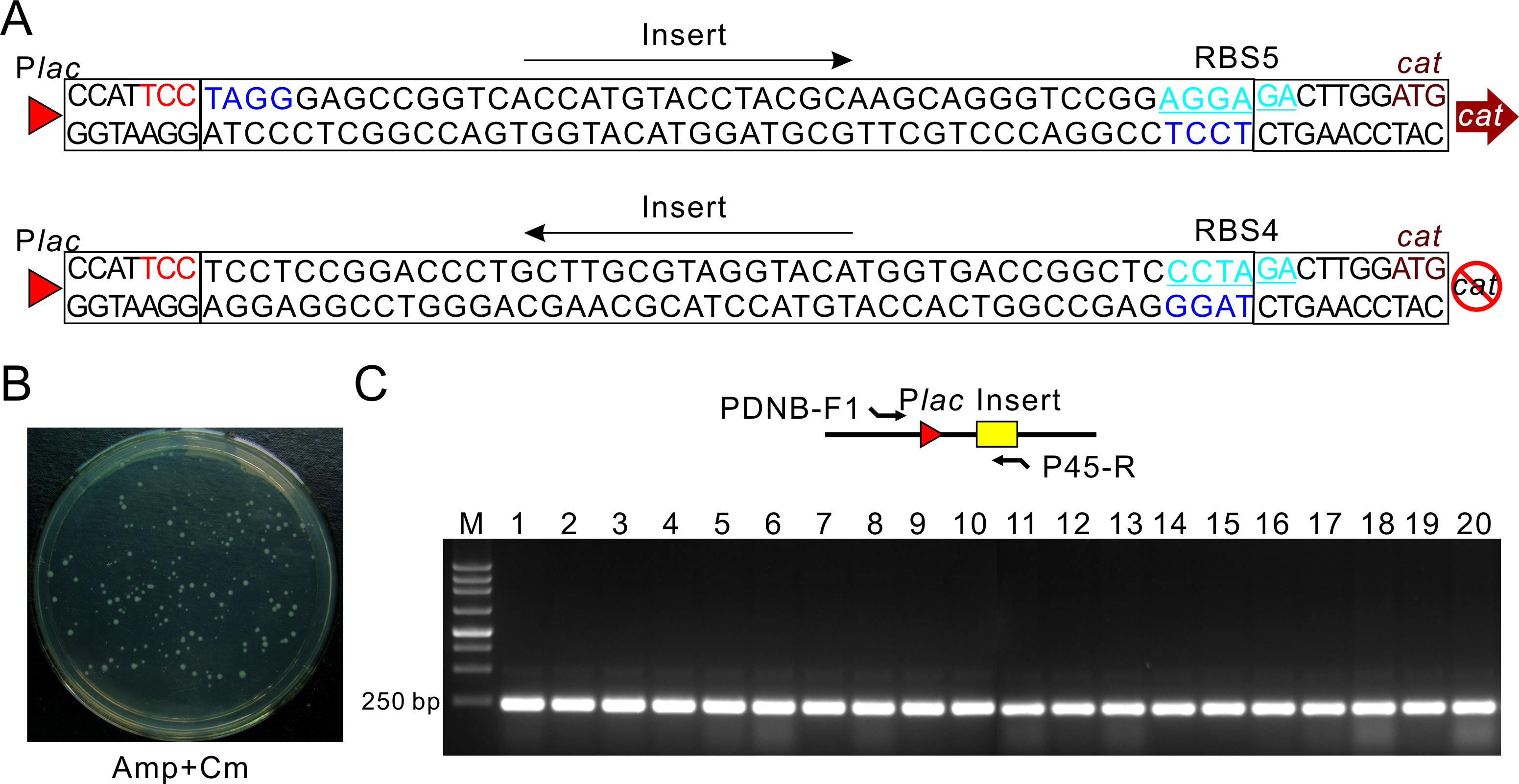

Supplement: Figure S4 — Application of pDNB100-B in cloning a 45 bp fragment. (A) RBS sequences formed by forward and reverse insertion of the 45 bp fragment. The arrows indicate the orientation of the inserts. The ATG start codon for the cat gene is shown in brown. (B) Transformants of ligated products on plate with Cm. (C) PCR confirmation of colonies as shown in B. Paired positions for primers used in PCR are shown in the upper panel. (TIF) [file pone.0050142.s004.tif]
