# Supplementary material for: Ribosomal Binding Site Switching: An Effective Strategy for High-Throughput Cloning Constructions
Source: PLoS One. 2012 Nov 21;7(11):e50142. doi: 10.1371/journal.pone.0050142 (PMC3503710; doi:10.1371/journal.pone.0050142)
Supplement: Table S1 — Oligonucleotides used in this study. (DOC) [file pone.0050142.s005.doc]

**Table S1**. Oligonucleotides used in this study.

| **Name** | **Sequence (5’- 3’)** |
| --- | --- |
| PRBS1-cat-F | TAGGTCGACTTCCGATCTGGATGGAGAAAAAAATCACTGG |
| PRBS2-cat-F | TAGGTCGACTCCTGATCTGGATGGAGAAAAAAATCACTGG |
| PRBS3-cat-F | TAGGTCGACTCCAGATCTGGATGGAGAAAAAAATCACTGG |
| PRBS4-cat-F | TAGGTCGACCCTAGATCTGGATGGAGAAAAAAATCACTGG |
| PRBS5-cat-F | TAGGTCGACAGGAGATCTGGATGGAGAAAAAAATCACTGG |
| Pcat-KR | ATTGGTACCTTACGCCCCGCCCTGC |
| Pcat-BF | GCGAGATCTGAGAAAAAAATCACTGG |
| PDNB-KF | AAAGGTACCTTAAGCCAGCCCCGACACC |
| PDNB-BR | TTTAGATCTAAGCTTGAATTCTCCACACAACATACGAGCC |
| PccdB-EF | CCGGAATTCAAATAATTTTGTTTAACTTTAAGAAGGAGATATACATATGCAGTTTAAGGTTTACACCTAT |
| PccdB-KR | CGTGGTACCTTATATTCCCCAGAAC |
| PXcmI-EF | AGCGAATTCCCATTCCTAAGCTGGGTCTCATGAGCGGATACATATT |
| PXcmI-BR | TTAAGATCTCATCCAAGTCTGGAATGGTTCCCGACTGGAAAGCGGGC |
| POF | CAAAATTATTTGGAGTCTCCACACAACATACGAG |
| POR | GGAGACTCCAAATAATTTTGTTTAACTTTAAG |
| PlacZA-TF | AGGGAAGGAGATATACATATGACCATGATTACGGATTC |
| PlacZA-TR | CCTTCAGACGACAGTATCGGCCT |
| Pgfpuv-TF | AGGGAAGGAGATATACATATGGCTAGCAAAGGAGAAGA |
| Pgfpuv-TR | CCTTTATTTGTAGAGCTCATCCATG |
| PlacZA-BF | TAGGGAAGGAGATATACATATGACCATGATTACGGATTC |
| PlacZA-BR | TCCTTCAGACGACAGTATCGGCCT |
| Pgfpuv-BF | TAGGGAAGGAGATATACATATGGCTAGCAAAGGAGAAGA |
| Pgfpuv-BR | TCCTTTATTTGTAGAGCTCATCCATG |
| PlacZA-101-BF | AGGATGACCATGATTACGGATTC |
| Placp-106-F | CCTCTCTTCAAATAATTATATCACAGCAACGCGGCCTTTTTACGGTTC |
| Placp-106-R | AGGCTTTGGAAAAGGGAGCCAAAGG |
| P45-F | TAGGGAGCCGGTCACCATGTACCTACGCAAGCAGGGTCCGGAGGA |
| P45-R | TCCTCCGGACCCTGCTTGCGTAGGTACATGGTGACCGGCTCCCTA |
| PDNB-F1 | CGGACTAGTTGTGGATAACCGTATT |
